# Renal denervation: a glimpse of hope?

**DOI:** 10.1007/s12471-017-0998-8

**Published:** 2017-05-04

**Authors:** M. Voskuil

**Affiliations:** Department of Cardiology, University Medical Center Utrecht, Utrecht, The Netherlands

The development of the treatment modality using renal denervation with radiofrequency energy endured many ups and downs [1]. After encouraging results in the initial studies, a pronounced variability in blood pressure responses of individual patients was noted in clinical practice. Trying to unravel this observed variability, several technical shortcomings, in addition to the issue of selecting the appropriate patient population, were distinguished. For example, the induced lesions had a restricted distribution and limited penetration depth, leaving a large part of the nerves in the perivascular areas distant from the vascular lumen unaffected [2]. This may be due to the increased intima and media thickness in these hypertensive patients. Furthermore, the initial advice to perform ablations primarily in the main renal artery, leaving the distal segments and side branches untreated, may not have been good advice, in hindsight. Increased interest in the anatomy of sympathetic renal innervation has led to several novel insights [3]. For example, there is a high interpatient variability in the anatomy of these nerves. However, in general, the density of renal sympathetic nerves is higher and the location of these nerves more superficial in the distal segments. Also, in renal arteries with larger diameters and thicker vessel parenchyma, the innervation is found further from the lumen and the nerves increase in thickness. These findings suggest that the ablation lesions might have been insufficient with respect to location, depth and, most likely, also in number, in many cases in the initially performed procedures.

The concept of performing a redo procedure in patients who are non-responders to the initial treatment strategy seems reasonable in the light of the above-mentioned limitations. In their manuscript in this issue of *The Netherlands Heart Journal, *Daemen et al. describe a case series of three consecutive non-responders who underwent a repeat-procedure with a second-generation multi-electrode radiofrequency catheter [4]. The procedure was performed after an average of 22 months following first treatment. These patients showed a decrease in office-based and ambulatory blood pressure of −27/−6 mm Hg and −15/−13 mm Hg, respectively, after this second treatment. Notably, no arterial damage was observed after a follow-up of six months. Therefore, Daemen et al. conclude that a redo procedure using this second-generation system, and with new insights in mind, seems safe and might be of additional value in these difficult-to-treat patients.

Although the idea of performing a redo procedure in this situation is appealing, the results of this study must be considered with caution. First, the manuscript describes a limited experience in only three patients. Second, the catheters that were used during the index procedure were second-generation devices, compared to the Symplicity Flex system in the initial era of renal denervation. Initially, the patients did not respond to treatment with either a multi-electrode system (Vessix V2) or a circumferential ablation technique (Paradise and OneShot system). With regard to some of these technical aspects, the EnligHTN system appears to be similar to the systems that were used in the first procedure. Third, the patients were ablated only in the proximal segment of the renal arteries, as described by the authors, in both the first and the second procedure. This is theoretically one of the disadvantages of the EnligHTN system; it is too bulky to enter smaller sized distal or side branches. Remarkably, the patients still responded better to the second treatment. Could this be because a higher number of ablations in total after two procedures increases the chance of targeting enough nerve bundles to show an effect on blood pressure? Or is this system somehow more potent, i. e. capable of deeper ablations, than the other systems? The authors state, with good reason, that they could not exclude that the redo procedure might have had a similar effect if devices other than the EnligHTN system would have been used. To answer this question, a study with a direct head-to-head comparison of the different devices should be performed.

With the potential shortcomings in mind, also of the available second-generation devices, it remains of the utmost importance that a read-out for renal denervation is developed. A few small studies have evaluated different techniques for this reason. Some of the results showed that renal stimulation-induced blood pressure changes were correlated with changes in ambulatory blood pressure measurements after renal denervation [5]. Therefore, the authors stated that this technique might predict the response to renal denervation and, as a consequence, could serve as a read-out. Also, other techniques, using, for instance, invasively assessed renal artery haemodynamics, might be of use in future studies.

Recently, the long-awaited results of the Dutch multicentre Sympathy trial were published [6]. Unfortunately, these results showed that, as with HTN-3, the effects of renal denervation were not superior to standard care. The researchers showed that changes over time in medication adherence were common and affected treatment estimates considerably. Therefore, the currently running Spyral HTN OFF-MED/Spyral HTN ON-MED, which includes patients without medication or with a firm regime of confirmed medication use, will be pivotal. If this study (and several others running right now) does not show positive results over the coming 12 months, a definite loss of belief in this treatment modality is difficult to avoid.

In conclusion, the paper by Daemen et al. is hopeful, but as Aristotle already stated long ago: ‘*One swallow *(or in this case, three)* does not make a summer*’ [4].
